# The Treatment of Alveolar Abscess

**Published:** 1905-03-15

**Authors:** J. P. Buckley


### THE TREATMENT OF ALVEOLAR ABSCESS.

BY J. P. BUCKLEY, PH. G., D.D.S.,

In our last article we considered in detail the treatment of chronic abscesses of the blind variety, and stated that, in those cases where the pus continues to flow freely when the dressing is removed at the third or fourth sitting, some complication can be expected, in which case it is useless to attempt further treatment by the ordinary method. Many of these stubborn cases will yield nicely to our treatment after gently forcing some stimulating agent, such as a 10 percent solution of trichoracetic acid or a 25 or 50 percent solution of phenol-sulphonic acid, through the apices of the roots, having mechanically evacuated the pus previous to the use of these agents. Occasionally, however, it is necessary to surgically establish an opening through the overlying process and soft tissue and treat as an ordinary discharging abscess, which treatment will now be considered.

It is our first duty in treating these cases to locate the offending tooth. Ordinarily this is a simple matter, for the reason that the sinus usually opens immediately over the tooth from which it comes. The pus in making its exit, however, follows the line of least resistance, and in some cases the condition of the process is such that the pus burrows forward or backward and opens through the gum at a point several teeth removed from the one which is causing the trouble. These are the cases that are difficult to diagnose, especially where the abscess has been discharging for some time, when there is not much tenderness in any special tooth and where there are several devitalized teeth on this particular side of the mouth. Sometimes two teeth containing putrescent pulps have a common fistula. In this case it would be impossible to heal the tract by treating only one of the teeth. The use of a silver probe will be valuable in all of these cases. By gently working the probe forward and backward, as the case may be, the sinus can be explored and the offending tooth or teeth located without drilling into innocent teeth—a discouraging procedure, both to the patient and to ourselves. The history of the case, as given by the patient, will often be of service, but judgment may be exercised and due allowance made. Our greatest difficulty, then, in treating many of these chronic discharging abscesses will be had in locating the offending tooth. This accomplished, the rest is easy.

The method of applying drugs to the treatment of these cases is so well known that it appears almost a waste of time and space for me to attempt to describe it. All that is necessary to effect a cure, there being no complication, is to force some *bland solution* through the root canal and fistula—thus being certain, it is well established, cauterize the tract, hermetically seal in the canals the same agent used for this latter purpose, and at a subsequent sitting fill the canals.

Frequently difficulty is encountered in establishing the fistula. For this purpose I use, in a large hypodermic syringe, an essential-oil water, to which two minims of carbolic acid have been added for every fluid-ounce of the solution. With the anterior teeth a long, straight needle is best, while a curved needle is necessary with the posterior. If the gum has been previouly lanced over the end of the root, dipping the lancet in carbolic acid, and the needle placed well up or down into the canal, and the gutta-percha or vulcanite rubber packed tightly around the needle into the cavity and held in place, the solution can quite readily be forced through the entire fistulous tract. There are two objects in forcing this bland solution through the fistula:

one is to be certain that it is open and the other is to mechanically wash out the pus. Wherever pus can be mechanically removed it is always better to dispose of it by this means rather than to attempt to do so by the use of some chemical agent. I know it is the common practice, after the fistula is established by the use of some bland solution, to follow with hydrogen dioxide. This is often a dangerous procedure and *always unnecessary* if the first solution has been used properly and in sufficient quantity. There is no advantage in using an essential-oil water, except, in my office, it is always fresh, sterile and convenient, and it is also pleasant to the patient. After a considerable quantity has been forced through the fistula and it is well established, the canals should be dehydrated with alcohol and warm air and 95 percent carbolic acid placed in the canals on cotton (never use a syringe for this purpose), and with gutta-percha or vulcanite rubber and a suitable instrument produce gentle pressure until the mouth of the fistula is cauterized, having alcohol ready to neutralize any of the carbolic acid that may escape in the patient's mouth. The rubber or gutta-percha and cotton can now be removed (being certain that all of the cotton is *removed*), when a dressing of carbolic acid should be hermetically sealed in the canals from three days to a week, at which time, if the fistula is healing nicely and the case gives a favorable history, the root, in my opinion, should be filled. I do not believe in delaying the root-filling very long after the fistula has been cauterized, for by filling the root as soon as we are certain that the fistula is healing we can avoid a weeping condition which usually exists when this part of the treatment has been delayed for one month or six weeks.

In treating chronic abscesses that have been discharging for a considerable length of time, and where we can reasonably suspect a roughening of the end of the root or process through which the pus has been discharging, it is the best practice to substitute *pure phenol-sulphonic acid* for the carbolic acid which is generally used. In using phenolsulphonic acid for this purpose care most be taken not to use too much and to avoid getting any of the remedy on the patient's face or clothing. Inasmuch as this agent rapidly disintegrates cotton, it should be applied to the canals on shreds of asbestos or silk. I want to emphasize the use of phenol-sulphonic acid in the treatment of all of these complicated alveolar abscesses. This agent not only cauterizes the fistulous tract, but it chemically dissolves any sharp edges, which may be a source of irritation, and prevent the healing of the fistula. Where these blind and discharging abscesses will not yield to the treatment herein outlined it is necessary to further assist nature by surgical procedures, such as the amputation of the root, etc. In these articles it has been my object to discuss the application of drugs and remedies to the treatment of various diseased dental conditions. I shall not attempt here to discuss the surgical methods employed.

In my opinion an operation is not a success whereby the dentist has treated a putrescent pulp, a blind or discharging abscess, and filled the root and crown of the tooth, if the *color* of the tooth structure of the crown has not been restored, inasmuch as the color of the teeth in these cases is usually lost before the patient presents himself for treatment.—*Review.*

